# Timely enteral nutrition of ventilated polytrauma patients: current standards and room for improvements

**DOI:** 10.1007/s00068-025-02849-z

**Published:** 2025-04-09

**Authors:** Ottavio de la Vega, Saskia Ridley-Smith, Howard Huang, Daniel Hali, Simone Meakes, Cino Bendinelli, Zsolt J. Balogh

**Affiliations:** 1 School of Medicine and Public Health, University of Newcastle, Newcastle, NSW Australia; 2 Department of Traumatology, John Hunter Hospital, Newcastle, NSW Australia; 3 Discipline of Surgery, School of Medicine and Public Health, University of Newcastle, Callaghan, NSW Australia; 4 Injury and Trauma Research Program, Hunter Medical Research Institute, Newcastle, NSW Australia

**Keywords:** Polytrauma, Enteral nutrition, Intensive care unit, Trauma

## Abstract

**Purpose:**

Polytrauma patients in intensive care units (ICUs) face significant risks of morbidity and mortality, with nutrition playing a crucial role in mitigating energy deficits and complications such as multi-organ failure (MOF). This study aimed to evaluate adherence to enteral nutrition (EN) guidelines in ventilated polytrauma patients and explored correlations between EN timing and clinical outcomes.

**Methods:**

A four-year retrospective (2019–2022) analysis of ventilated polytrauma patients (abbreviated injury scale > 2 in ≥ 2 body regions) admitted to a level 1 trauma centre. Collected data included demographics, injury characteristics and EN patterns. Early EN was defined as started withing 24 h. Statistical analysis assessed associations between EN, injury severity, and outcomes such as ICU length of stay (LOS), mortality, and MOF.

**Results:**

Of 182 patients (median age 41, male 77%, median ISS 34), 41 did not receive EN and were excluded. Of the remaining 141, 64% received early EN, with a median time to EN of 17.8 h. Early EN was associated with reduced ICU LOS (*p* = 0.016). Delaying EN initiation correlated with higher injury severity (*p* = 0.008). Each one-hour delay to EN increased MOF odds by 1.47% (OR: 1.0147, *p* = 0.07). EN interruptions (> 6 h) occurred 354 times.

**Conclusion:**

Investigations into current EN standards in polytrauma patients demonstrated an average of 2.5 interruptions in EN exceeding 6 h per patient, with 40% not fed within 24 h. Combined with inconsistent dietician input, this offers room for improvement as early EN is associated with better outcomes, with a reduced ICU LOS established through this study.

## Introduction

Polytrauma, defined as severe injury of two or more body regions, is associated with significant morbidity and mortality. These severely injured patients typically require several surgical procedures, a prolonged intensive care unit (ICU) and hospital stay and a lengthy rehabilitation [1]. They are also at risk of developing multiple organ failure (MOF) (18 (9.8%)). In a 13-year retrospective analysis, 27% of severe trauma admissions were defined as polytrauma [2].

Polytrauma causes a state of hyperinflammation; which is a metabolically demanding and energy intensive process. Nutrition is considered an essential modifiable factor to minimise this negative caloric balance [3]. These patients are often mechanically ventilated and sedated and enteral nutrition (EN) is provided via nasogastric tube (NGT) or nasojejunal tube (NJT), delivering to the stomach or past the pylorus, respectively.

Early EN, defined as initiation within 24 h from admission to ICU, is recommended by most guidelines for critically ill patients [4, 5, 6, 7]. The non-polytrauma specific EN quantity and quality can be defined for the critically ill population by using the suggested equation of 25–30 kcal/kg/day, and protein requirement of 1.2–2.0 g/kg/day [4].

## Aim

The aim of this study was to evaluate adherence to enteral nutrition guidelines in polytrauma patients admitted to ICU. The correlation between timing to EN and clinical outcomes was also investigated.

## Methods

A four-year retrospective study ending on 31st December 2022 was performed on ventilated polytrauma patients admitted to a Level 1 Trauma Centre ICU. The institutional trauma registry was queried for: polytrauma patients (defined as Abbreviated Injury Scale (AIS) > 2 in two or more body regions), older than 16 years and admitted to the ICU on mechanical ventilation. Patients who did not receive EN during ICU admission, those who were discharged or died within 48 h from admission were excluded. Clinical data beyond the trauma registry was collected from the electronic Record for Intensive Care (eRIC) and patient files.

### Collected variables

Collected patient characteristics included: age, sex, mechanism of injury, Injury Severity Score (ISS) and AIS of all body regions. The worst vital signs in the emergency department were collected (heart rate, systolic blood pressure, and Glasgow Coma Scale). Laboratory tests included: on arrival serum lactate and haemoglobin concentrations. Blood products transfused within 24 h of injury were also recorded.

Nutritional data included: hours from ICU admission to EN, feeding route, dietician input, time to nutritional goal, and interruptions to EN (longer than 6 h). EN interruptions were categorised by causation: ileus, airway event (e.g. extubation planning or attempt), ICU diagnostics or procedures (e.g. transoesphageal echocardiogram, endoscopy), procedures outside the ICU (e.g. imaging, surgery), accidental NGT removal, and undetermined (reason was not identified). Surgical procedures were categorized into craniotomy/craniectomy, laparotomy with and without bowel resection, damage control laparotomy, orthopedic procedures, and others. Disposition from hospital (home, nursing facility, acute care facility, rehabilitation facility, or death), as well as complications during ICU admission, (ventilator-acquired pneumonia (VAP), sepsis, ileus and vomitus) were also collected. Severe traumatic brain injury (TBI) was defined as AIS Head ≥ 4 and initial GCS ≤ 9. Severe abdominal trauma was defined as AIS abdomen ≥ 4.

### Outcome measures

Primary outcome was time to EN initiation. Secondary outcomes included: EN interruptions, dietician involvement, time to reach nutritional goal, ICU and hospital LOS, mortality, MOF, complications.

### Statistical analysis

Subgroup analysis was performed between patients who received early EN (< 24 h from admission to ICU) and late EN (> 24 h).

Normality of distribution was determined with Shapiro-Wilk test. While categorical variables were tested with Chi-Square, continuous variables were tested with Wilcoxon-Mann–Whitney test.

A logistic regression model was used to calculate the odds of developing multi organ failure (MOF) given a change in time to EN. Furthermore, as ISS is not a true continuous variable, the appropriateness of using linear regression model was assessed using model residual diagnostics and the assumption was deemed appropriate. The association between ISS as a continuous variable and time to EN was tested with a linear regression model. Statistical significance was set at p-values < 0.05.

## Results

**Fig. 1:**
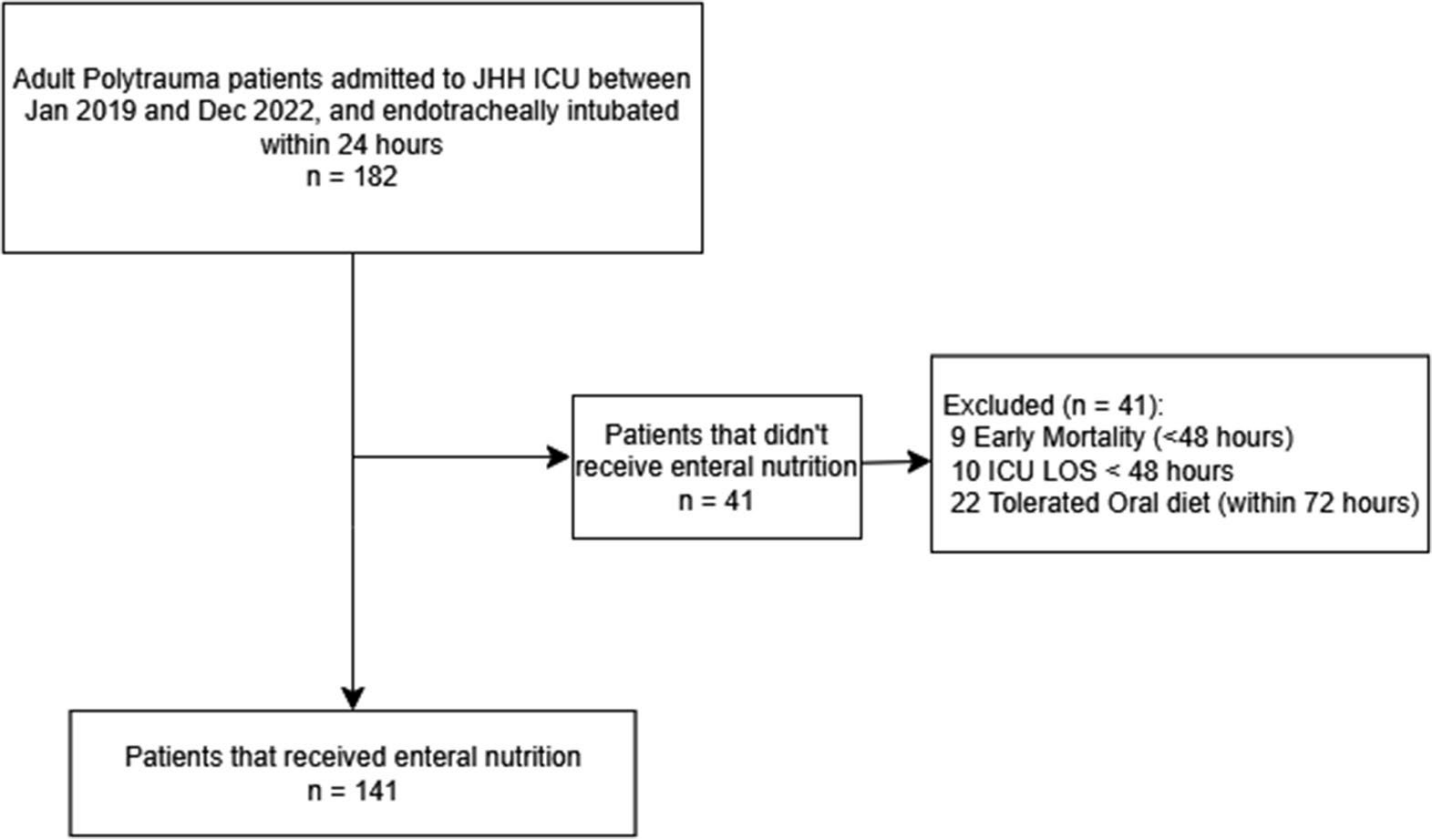
Patient flowchart. Flowchart of patients receiving enteral nutrition. ICU = Intensive Care unit; LOS = Length of stay

A total of 182 patients met inclusion criteria. Upon chart review, 19 patients were excluded owing to early mortality or discharge within 48 h of admission. A further 22 patients were excluded due to early extubation and ability to commence on oral diet without requiring consideration for EN (Fig. 1).

Table 1 describes the entire cohort of 182 polytrauma patients. Males represented 77% of the cohort and median age was 41 (25–61). Blunt trauma was the most common mechanism of injury (94%). The median worst heart rate and blood pressure values on arrival were 93 (78–112) and 102 (91–117) respectively, with a median worst on arrival lactate of 2.6 (1.7–4.6).

**Table 1:** Demographics and clinical data of 182 polytrauma patients admitted to ICU and ventilated

Variables	Measure
Age: median (IQR)	41 (25–61)
Male: n (%)	141 (77.5)
Worst systolic BP on arrival: median (IQR)	102 (91–117)
Worst heart rate on arrival: median (IQR)	93 (78–112)
Red blood cells transfused within 24 h: median (IQR)	5 (3–7)
Plasma transfused within 24 h: median (IQR)	2 (2–5)
Cryoprecipitate administered within 24 h: median (IQR)	5 (4–5)
Platelets transfused within 24 h: median (IQR)	1 (1–2)
GCS: median (IQR)	3 (3–3)
Lactate: median (IQR)	2.6 (1.7–4.6)
Base Excess: median (IQR)	-3.1 (-6-0.9)
Blunt Trauma: n (%)	171 (94)
Severe TBI (AIS Head ≥ 4 & initial GCS ≤ 9): n (%)	91 (50)
Severe abdominal trauma (AIS Abdomen ≥ 4): n (%)	25 (13.7)
ISS: median (IQR)	34 (27–43)
Days in ICU: median (IQR)	7 (3–13)
Days in hospital: median (IQR)	22 (10–39)
Craniotomy / Craniectomy: n (%)	31 (17)
Laparotomy with bowel resection: n (%)	5 (2.7)
Laparotomy without bowel resection: n (%)	7 (3.8)
Damage control and open abdomen: n (%)	11 (6)
Orthopaedic procedure: n (%)	87 (47.8)
Other procedures: n (%)	34 (18.7)
Ventilator-acquired pneumonia: n (%)	31 (17)
Sepsis: n (%)	17 (9.3)
In-hospital mortality: n (%)	25 (13.7)
Home: n (%)	26 (14.3)
Rehabilitation facility: n (%)	71 (39)
Acute care facility: n (%)	38 (20.8)
Nursing facility: n (%)	22 (12)
EN: n (%)	141 (77.5)
TPN: n (%)	3 (1.6)
No EN: n (%)	41 (22.5)

IQR = interquartile range; ICU = Intensive Care unit; ISS = Injury Severity Score; AIS = Abbreviated injury scale; TBI = Traumatic brain injury; EN = Enteral Nutrition; TPN = Total Parental Nutrition; GCS = Glasgow Coma Scale

**Fig. 2:**
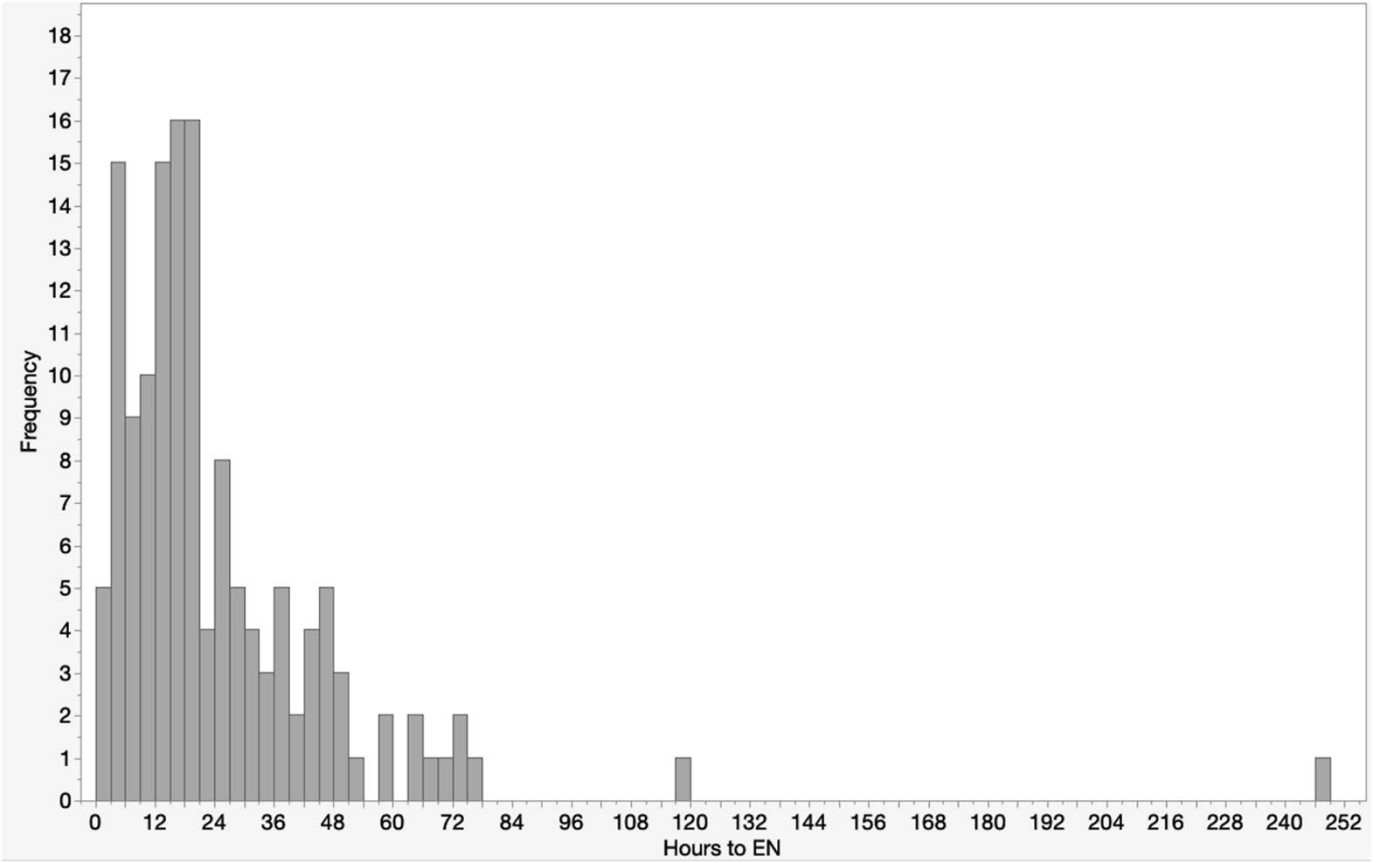
Time to initiation of enteral nutrition of polytrauma patients on the intensive care unit. Bar graph demonstrating hours to enteral nutrition initiation and frequency. EN = Enteral nutrition

**Fig. 3:**
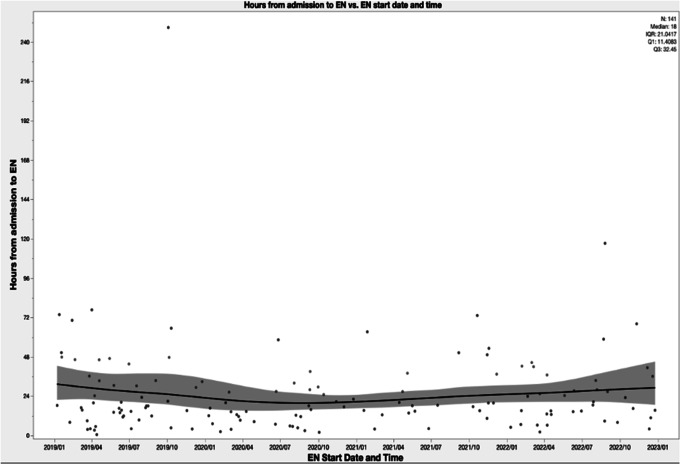
Time to EN in hours vs. date and time of EN administration. Scatter plot with trend line of hours to enteral nutrition initiation over study period. EN = Enteral nutrition

This was a cohort of very sick patients (median ISS 34), who required large amounts of blood products (median red blood cells received in 24 h was 5), and several surgical procedures (0.96 procedures per patient). Of these 50% suffered from severe TBI and 33% required neurosurgical intervention. Out of the 25 (13.7%) patients diagnosed with severe abdominal trauma, 52% of them required abdominal surgery. The most commonly, the study population patients had musculoskeletal operative procedures (48%). Median ICU and hospital LOS were 7 and 22 days, respectively. Mortality occurred in 14%. The most common disposition was rehabilitation facilities (39%) (Table 1). When included and excluded groups were compared, these differed in mechanism of injury, and ICU and hospital LOS (Table 2). After exclusion criteria were applied, 141 patients were selected for further analysis. Time to EN showed a skewed distribution to the left with the median time to EN of 17.8 h (Fig. 2), despite two outliers. The first outlier suffered a conservatively managed bowel obstruction, and the second outlier had a 5 day delay in EN, whilst also developing MOF. During the study period, median time to EN did not vary significantly (estimate − 0.003, *p* = 0.5545) (Fig. 3).

**Table 2:** Demographics and clinical data of included and excluded cohorts

Variables	Enteral nutrition received (*n* = 141)	No enteral nutrition (*n* = 41)	*P*-value
**Age: median (IQR)**	44.5 (23.75.5–63)	35 (25.5–51)	0.3693
**Male: n (%)**	106 (75)	35 (85.4)	0.1693
**Injury Characteristics**			
Blunt Trauma: n (%)	136 (96.5)	35 (85.4)	**0.0087**
Severe TBI (AIS Head ≥ 4 & initial GCS < 9): n (%)	72 (51)	19 (46.3)	0.5945
Severe abdominal trauma (AIS Abdomen ≥ 4): n (%)	19 (13.4)	6 (14.6)	0.8495
ISS: median (IQR)	34.5 (28.5–41)	34 (25–45)	0.9058
AIS Head & Neck: median (IQR)	4 (3–4)	4 (2.75-5)	
AIS Face: median (IQR)	2 (2–3)	2 (2–3)	
AIS Chest: median (IQR)	3 (3–4)	3 (3–4)	
AIS Abdomen: median (IQR)	3 (2–4)	3 (2–4)	
AIS Extremities: median (IQR)	3 (2–4)	3 (2–5)	
AIS External: median (IQR)	1 (1–1)	2 (1–3)	
**Days in ICU: median (IQR)**	9 (5–16)	2 (1–3)	**< 0.0001**
**Days in Hospital: median (IQR)**	25 (13.75-47)	10 (4-20.5)	**< 0.0001**
Multiple Organ Failure: n (%)	17 (12)	1 (2.4)	0.0694
In-hospital mortality: n (%)	16 (11.3)	9 (22)	0.0825
Home: n (%)	18 (12.7)	8 (19.5)	0.2772
Rehabilitation facility: n (%)	63 (44.6)	8 (19.5)	0.0036
Acute care facility: n (%)	26 (18.4)	12 (29.3)	0.1332
Nursing facility: n (%)	18 (12.8)	4 (9.8)	0.6028
EN: n (%)	141 (100)	N/A	
TPN: n (%)	3 (2.1)	N/A	
Dietician consult during ICU admission: n (%)	89 (63.1)	N/A	
Hours to EN: median (IQR)	17.8 (11.16–32.23)	N/A	
Nutritional goal reached: n (%)	71 (50.4)	N/A	
Ileus (> 200 ml aspirate): n (%)	20 (14.2)	2 (4.9)	0.1076
Vomitus: n (%)	14 (9.9)	N/A	

IQR = interquartile range; ICU = Intensive Care unit; ISS = Injury Severity Score; AIS = Abbreviated injury scale; TBI = Traumatic brain injury; EN = Enteral Nutrition; TPN = Total Parental Nutrition; GCS = Glasgow Coma Scale

Early and late EN groups were statistically similar in demographics (age, sex) (Table 3), ISS, mechanism of injury, and presence of severe TBI. ICU LOS was shorter in the early EN group (*p* = 0.016), while mortality and MOF did not statistically differ (Table 3). Only 3 (6%) of the 51 patients in the late EN group received total parental nutrition. Hours to EN was significantly different between the two groups (*p* < 0.0001).

**Table 3:** Demographics and clinical data comparison between patients who received early and late EN

Variables	< 24 h (*n* = 90)	> 24 h (*n* = 51)	*P* value
**Age: median (IQR)**	49 (24–65)	38 (23–59)	0.5408
**Male: n (%)**	70 (77.8)	36 (70.6)	0.3423
**Injury Characteristics**			
Blunt Trauma: n (%)	87 (96.7)	50 (98)	0.6372
Severe TBI (AIS Head ≥ 4 & initial GCS < 9): n (%)	46 (51.1)	26 (51)	0.9881
Severe abdominal trauma (AIS Abdomen ≥ 4): n (%)	6 (6.7)	7 (25.5)	0.1639
ISS: median (IQR)	34 (26–38)	38 (29–45)	0.0804
AIS Head & Neck: median (IQR)	4 (3–5)	4 (3–4)	
AIS Face: median (IQR)	2 (2–3)	2 (1–3)	
AIS Chest: median (IQR)	3 (3–4)	3 (3–4)	
AIS Abdomen: median (IQR)	2 (2–3)	3 (2–4)	
AIS Extremities: median (IQR)	3 (2–4)	3 (2–4)	
AIS External: median (IQR)	1 (1–1)	1 (1–2)	
**Days in ICU: median (IQR)**	8 (5–13)	11 (7–22)	**0.016**
**Days in Hospital: median (IQR)**	24 (12–47)	28 (14–47)	0.6171
**Multi Organ Failure: n (%)**	10 (11.1)	7 (13.7)	0.6469
**In-hospital mortality: n (%)**	8 (8.9)	8 (15.7)	0.2214
Home: n (%)	11 (12.2)	7 (13.7)	0.7972
Rehabilitation facility: n (%)	39 (43.3)	16 (47.1)	0.1618
Acute care facility: n (%)	19 (21.1)	6 (13.7)	0.1626
Nursing facility: n (%)	13 (14.4)	5 (9.8)	0.4275
VAP: n (%)	19 (21.1)	12 (23.5)	0.7390
Sepsis: n (%)	9 (10)	8 (15.7)	0.3191
Infection complication: n (%)	21 (23.3)	15 (29.4)	0.4264
EN: n (%)	90 (100)	51 (100)	
TPN: n (%)	0	3 (5.9)	0.0200
**Dietician consult during ICU admission**: n (%)	57 (63.3)	36 (70.6)	0.3824
**Hours to EN: median (IQR)**	13.4 (6.6–17.1)	39.1 (29.6–50.6)	**< 0.0001**
**Nutritional goal reached**: n (%)	44 (48.9)	26 (51)	0.8114
Ileus (> 200 ml aspirate): n (%)	13 (14.4)	7 (13.7)	0.9064
Vomitus: n (%)	10 (11.1)	4 (7.8)	0.5330

IQR = interquartile range; ICU = Intensive Care unit; ISS = Injury Severity Score; AIS = Abbreviated injury scale; TBI = Traumatic brain injury; EN = Enteral Nutrition; TPN = Total Parental Nutrition; GCS = Glasgow Coma Scale

On logistical regression, the association between time to EN and MOF was not statistically significant. For each one-hour delay to EN, the odds of developing MOF increased by 1.47% (OR: 1.0147, 95% CI 1.0003–1.0338, *p* = 0.07). Similarly, ISS was statistically associated with time to EN, with each ISS unit linked to 0.53 h delay to EN (*p* = 0.008, 95% CI, 0.14 ~ 0.91).

Dieticians were involved in 63% of admissions, but the calculated nutritional goal was reached only in 50%. A total of 354 EN interruptions occurred, an average of 2.5 per patient. Unplanned NGT removal (34%), followed by procedures outside the ICU (27%) were the most frequently occurring causes for interruption (Table 4).

**Table 4:** Nutritional data

Variables	Measure
Hours from admission to EN	
< 24 h: n (%)	90 (64)
> 24 h: n (%)	51(36.2)
ICU dietician consult: n (%)	89 (48.6)
Nutritional goal reached: n (%)	71 (50.3)
**Feeding complications**	
Ileus: n (%)	22 (15.6)
Vomitus: n (%)	14 (10)
Fasting periods (> 6 h): n	354
Fasting periods per patient: median (IQR)	2 (1–4)
**EN interruptions: n (%)**	354
Interrupted for ileus (> 200 ml aspirate)	20 (5.6)
Interrupted for airway event	28 (7.9)
Interrupted for ICU procedures	61 (17.2)
Interrupted for procedures outside ICU	97 (27.4)
Interrupted for NGT dislodgment	121 (34.2)
Interrupted for undetermined reason	51 (14.4)

IQR = interquartile range; ICU = Intensive Care unit; EN = Enteral Nutrition; NGT = Nasogastric tube

## Discussion

Nutritional support is crucial in the critically ill, including the severely injured polytrauma patient [8, 9, 10, 11, 12]. Adequate nutrition can reduce organ dysfunction and support the healing process [3]. Prompt EN is required in those who receive endotracheal ventilation. Guidelines recommend EN within 24 h from admission [4, 5, 6, 7], aiming to decrease these complications, ICU LOS and mortality.

In this retrospective study from a Level-1 trauma centre with more than 1700 trauma admissions per year, most ventilated trauma patients (64%) received EN within the recommended timeframe of 24 h from admission to ICU. This figure did not change over the 4-year study period. Similar rates of adherence to guidelines were observed in other studies [13]. This cohort consisted of severely injured and critically ill patients with a median ISS of 34, half of which sustained severe traumatic brain injuries, and a high proportion of which required surgical interventions. The in-hospital mortality rate for this cohort was nearly twice the average mortality rate for severe trauma (ISS > 12) in New South Wales [2].

There were no statistically significant differences between early and late EN groups demographics and clinical indicators (ISS, mechanism of injury and severe TBI), suggesting similarity between the groups.

Interestingly, despite early EN initiation, frequent more than six hours interruptions were observed. These interruptions could explain why in 50% of patients the nutritional goal wasn’t met. Even in this relatively small retrospective study the statistical association between ISS and time to EN was significant. While ISS is not modifiable and frequently unknown during ICU stay, the fact that time to EN was associated with the development of MOF offers a preventive opportunity for this deadly syndrome, which has no specific treatment. Our contemporary cohort can help inform future prospective interventional trials regarding sample sizes, as the power calculations performed can assist in determining the optimal sample size to adequately detect statical differences.

The majority of supporting evidence within international guidelines suggests that early EN is most likely beneficial to critically ill ICU patients. The guidelines are not specific to polytrauma patients. The American Society for Parenteral and Enteral Nutrition [4] nutritional guidelines cite multiple meta-analyses on trauma patients admitted to ICU [12, 14], with no specification of polytrauma within the included studies. Similarly, the European Society of Intensive Care Medicine guidelines [6] reference the same meta-analysis [12], with no mention of polytrauma within the guidelines. The European Society for Clinical Nutrition and Metabolism guidelines [7] cite polytrauma within search terms, yet no specific papers cited within the guidelines reference polytrauma. Our specification of polytrauma patients serves as a first step towards exploring the rooms for improvement in ICU nutrition admission, and potential preventative or therapeutic margins to be achieved within clinical practice. Furthermore, the determination of the severity of injury within this cohort emphases the importance in optimising all aspects of care for polytrauma patients, and the magnitude of the role nutrition can play.

### Limitations

The main limitation was the design of a retrospective, single-centre investigation. Interruptions to EN only greater than 6 h were recorded, however specifications within the categories were limited (e.g. procedures outside the ICU were not separated between surgery and imaging). Dietician consult was considered as all or nothing binary variable and the nutritional target was not further specified. These important confounders and potential outcomes warrant focused prospective study design. Our study was purely descriptive and was not powered to secondary clinical outcomes such as MOF.

## Conclusion

This contemporary retrospective cohort of very high-risk polytrauma patients identified that over one-third did not receive early enteral nutrition, and 40% were not reviewed by a dietician. Furthermore, half of those who received dietician input failed to meet nutritional goals. Considering the statistical association between the earlier timing of EN to short ICU LOS, along with a potential association with reduction in MOF incidence, targeted investment into optimisation of nutritional care of polytrauma patients and adequately powered prospective studies are warranted.

## Data Availability

No datasets were generated or analysed during the current study.
